# Cas Adaptor Proteins Coordinate Sensory Axon Fasciculation

**DOI:** 10.1038/s41598-018-24261-x

**Published:** 2018-04-16

**Authors:** Tyler A. Vahedi-Hunter, Jason A. Estep, Kylee A. Rosette, Michael L. Rutlin, Kevin M. Wright, Martin M. Riccomagno

**Affiliations:** 1 Neuroscience Program, Department of Molecular, Cell and Systems Biology, University of California, Riverside, CA 92521 USA; 2 Cell, Molecular and Developmental Biology Program, Department of Molecular, Cell and Systems Biology, University of California, Riverside, CA 92521 USA; 3 Vollum Institute, Oregon Health & Science University, Portland, OR 97239 USA; 4 Department of Biochemistry and Molecular Biophysics, Columbia College of Physicians and Surgeons, Columbia University, New York, New York, 10032 USA

## Abstract

Development of complex neural circuits like the peripheral somatosensory system requires intricate mechanisms to ensure axons make proper connections. While much is known about ligand-receptor pairs required for dorsal root ganglion (DRG) axon guidance, very little is known about the cytoplasmic effectors that mediate cellular responses triggered by these guidance cues. Here we show that members of the Cas family of cytoplasmic signaling adaptors are highly phosphorylated in central projections of the DRG as they enter the spinal cord. Furthermore, we provide genetic evidence that Cas proteins regulate fasciculation of DRG sensory projections. These data establish an evolutionarily conserved requirement for Cas adaptor proteins during peripheral nervous system axon pathfinding. They also provide insight into the interplay between axonal fasciculation and adhesion to the substrate.

## Introduction

Precise assembly of the peripheral somatosensory system involves migration of neural crest cells (NCCs) to coalesce into sensory ganglia and subsequent guidance of axonal projections from these newly formed ganglia. The NCCs that give rise to the dorsal root ganglia (DRG) originate from the dorsal spinal cord and migrate ventro-medially between the neural tube and rostral somite^1,2^. Upon reaching the presumptive DRG region, these neural progenitors coalesce and continue to proliferate before committing to a neuronal or glial fate^3,4^. The newly born sensory neurons then extend a central and a peripheral axon branch, acquiring the characteristic pseudounipolar morphology^5^. The resulting central projections traverse towards the spinal cord and enter the central nervous system (CNS) through the Dorsal Root Entry Zone (DREZ), while the distal processes navigate long distances to innervate their peripheral targets^6,7^. Accurate guidance and fasciculation of these axons requires an intricately choreographed array of signaling cues acting on their cognate receptors^8,9^. Although much is known about the ligand-receptor pairs required for axon trajectories, very little is known about the cytoplasmic effectors that allow these axons to respond to guidance cues.

Cas signaling adaptor proteins mediate a variety of biological processes including cell migration and changes in cell morphology^10^, and exhibit specific expression patterns during neural development in rodents^11^. Cas proteins interact with various classes of signaling proteins, including cytosolic tyrosine kinases (like Src and Fak). Upon phosphorylation, Cas proteins can provide docking sites for SH2-containing effectors, including Crk, which stimulate Rac1-mediated actin remodeling^12^. We have recently uncovered an essential role for Cas family members during retinal ganglion cell migration^13^, yet our current understanding of Cas adaptor protein function during mammalian axon pathfinding *in vivo* is limited. One member of this family, p130Cas, has been proposed as a required downstream component of netrin-mediated commissural axon guidance in the chicken spinal cord^14^. Interestingly, Drosophila Cas (dCas) has been shown to participate downstream of integrin receptors in axon fasciculation and guidance of peripheral motor axons^15^. Whether Cas proteins play similar roles during mammalian peripheral nervous system (PNS) development is currently unknown.

Here we examine the requirement for Cas adaptor proteins during DRG axon pathfinding. Our genetic data supports a novel role for Cas adaptor proteins during the fasciculation and guidance of central DRG projections in the DREZ. These data provide insight into the interplay between adhesion to the substrate and axon fasciculation.

## Results

**Figure 1:**
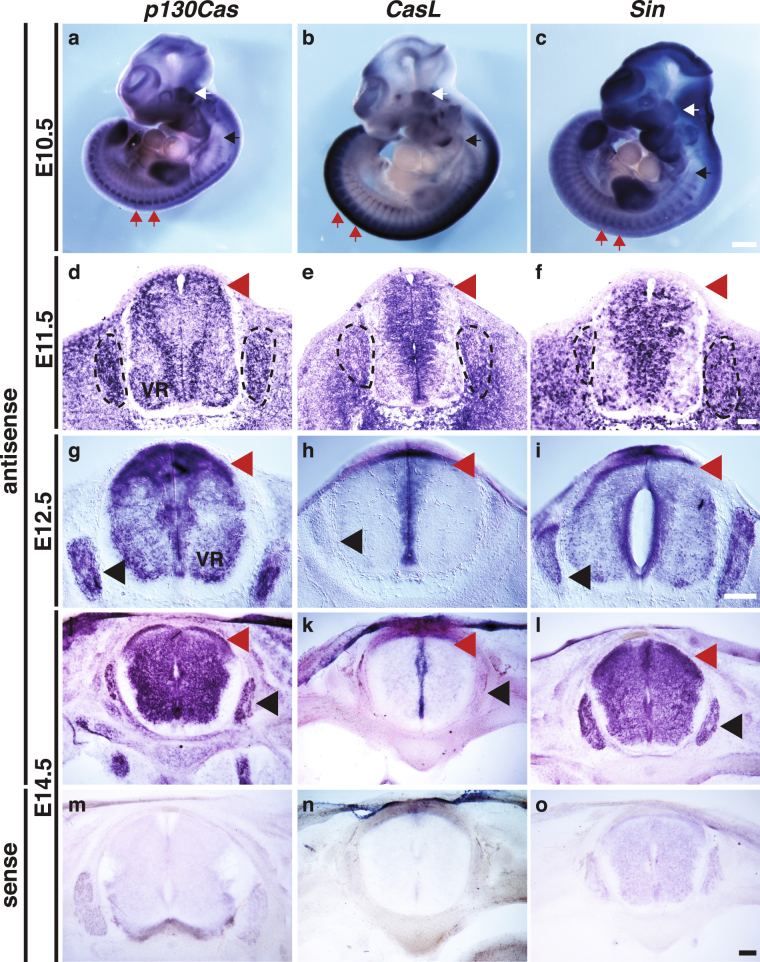
Expression of *Cas* genes in the developing dorsal root ganglia and spinal cord. **(a**–**c)** Whole-mount *in situ* hybridization for p*130Cas*, *CasL* and *Sin* in e10.5 embryos. White arrows mark the trigeminal ganglion, black arrows mark the nodose/petrosal compex, and red arrows point at DRG examples. **(d**–**o)** Transverse sections through embryonic spinal cords stained by *in situ* hybridization with probes against p*130Cas* (**d**,**g**,**j**,**m**), *CasL* (**e**,**h**,**k**,**n**) and *Sin* (**f**,**i**,**l**,**o**), at e11.5 (**d**–**f**), e12.5 (**g**–**i**), and e14.5 (**j**–**o**). *Cas* genes show overlapping expression in the DRGs (dotted line and black arrowhead) and dorsal spinal cord (red arrowheads). No staining was detected for the sense probes (**m**–**o**). VR: ventral roots. Scale bars: 500 μm for (**a**–**c**); 100 μm for (**d**–**f**); 200 μm (**g**–**i**) and 100 μm (**j**–**o**).

Cas adaptor proteins have been shown to participate in the formation of the neuronal scaffold of the mammalian retina^13^. In addition, dCas is required for integrin-mediated peripheral axon guidance and fasciculation in Drosophila^15^. To investigate the role for Cas signaling adaptor proteins during mammalian PNS axon guidance, we started by assessing the expression pattern of *Cas* genes during embryonic development by *in situ* hybridization (Fig. 1a–l). *Cas* genes are broadly expressed in the DRG, but display specific regional expression in spinal cord (SC) at embryonic day (e)10.5 and e11.5 (Fig. 1a–f; Supplementary Fig. S1a–c). *p130Cas* is mainly expressed in the mantle zone, with high levels of expression in the dorsal SC and ventral root (Fig. 1d). *CasL* expression in the spinal cord is primarily found in the dorsal SC and ventricular zone (Fig. 1e). *Sin/EFS* is also expressed around the ventricular zone of the SC, and subsets of DRG neurons (Fig. 1f). *p130Cas* and *Sin* continue to be expressed in the DRG at e12.5 and e14.5 (Fig. 1g,i,j,l). *CasL* DRG expression becomes weaker as development progresses, and becomes undetectable by e14.5 (Fig. 1b,e,h,k). All three *Cas* family members maintain expression in the spinal cord until at least e14.5 (Fig. 1d–l). At e12.5, *p130Cas* maintains expression in the mantle zone, dorsal SC and ventral root, but also begins to be expressed in the ventricular zone (Fig. 1g). A similar expansion in expression domain is observed for *Sin* (Fig. 1i). By e14.5 *p130Cas* and *Sin* appear to be broadly expressed in the SC, with stronger expression in the DREZ and ventral roots (Fig. 1j,l). At e12.5 and 14.5, *CasL* expression remains restricted to the ventricular zone (Fig. 1h,k). Sense negative control probes displayed negligible staining (Fig. 1m–o; Supplementary Fig. S1d–f).

**Figure 2:**
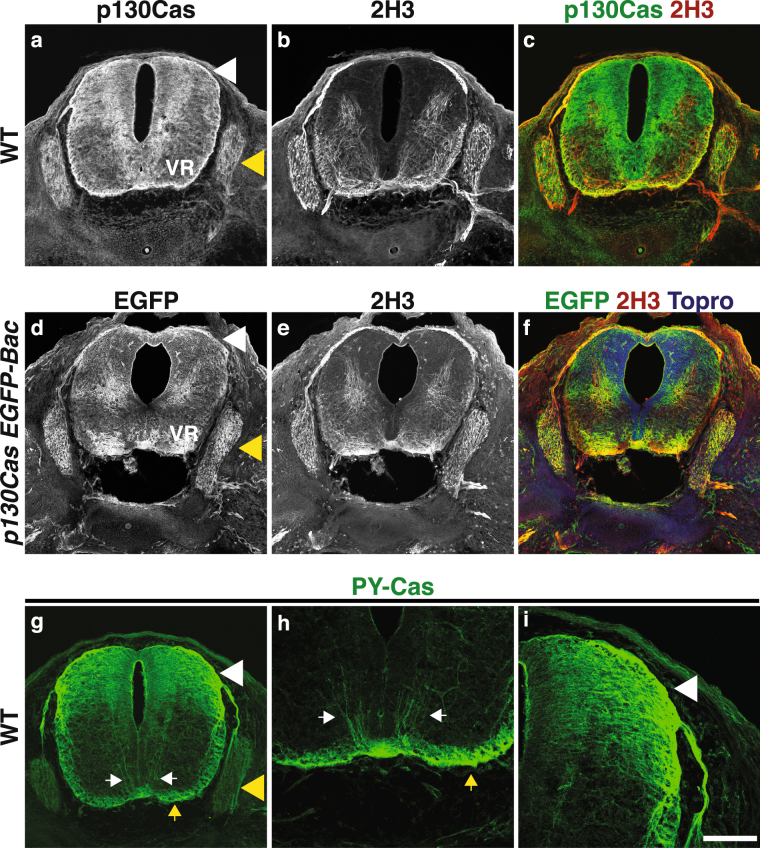
p130Cas is phosphorylated in commissural axons and DRG central projections. (**a**–**c**) Expression profile of p130Cas protein (green) in transverse sections through the mouse spinal cord at e12.5. Anti-Neurofilament (2H3, red) was used to reveal axons. (**d**–**f**) Immunofluorescence for EGFP (green) and 2H3 (red) in e12.5 *p130Cas EGFP-Bac* spinal cords. ToproIII (blue) was used to counterstain nuclei. (**g**–**i**) Expression of a phosophorylated-p130Cas (PY-Cas, green) in e12.5 spinal cord and DRGs. (**h**) PY-Cas is present in the ventral funiculus and commissural axons. (**i**) p130Cas phosphorylation is mainly found in the dorsal spinal cord and is enriched in DRG axons and DREZ. White arrowheads: DREZ; Yellow arrowheads: DRG; White arrows: commissural axons; Yellow arrows: ventral funiculus; VR: ventral roots. Scale bar: 200 μm for (**a–g**) and 100 μm for (**h**–**i**).

We next performed histological analyses of p130Cas protein expression in the developing spinal cord and DRG (Fig. 2a–c; Supplementary Fig. S2). The expression pattern of p130Cas protein overlaps with that of *p130Cas* mRNA in DRG and spinal cord mantle zone cell bodies (Figs 1g, 2a–c, Supplementary Fig. S2a–d). In addition, p130Cas protein localizes to DRG central projections and spinal cord commissural axons, and is highly enriched in the DREZ and dorsal funiculus (Fig. 2a, Supplementary Fig. S2a,c). Although there are some modest differences in the p130Cas mRNA and protein expression patterns inside the SC, these are likely due to the distinct subcellular distribution of the protein. The overall pattern of expression was confirmed by utilizing a GENSAT BAC transgenic mouse line that expresses enhanced GFP (EGFP) under the control of *p130Cas* regulatory sequences (Fig. 2d–f, Supplementary Fig. S3a,b,d,e,g,h)^16,17^. This transgenic line allows for the detection of cells expressing *p130Cas*^13^. The *p130Cas-EGFP-Bac* spinal cord EGFP expression pattern is consistent with that of endogenous p130Cas protein and mRNA in wild-type (WT) animals, with strong signal in dorsal SC, ventral root and DRG (Fig. 2d–f, Supplementary Figs S2, S3). As expected, no EGFP detection is observed in WT animals (Supplementary Fig. S3c,f,i). As phosphorylation of Cas adaptor proteins mediates adhesion signaling during neural development^13,15,18^, we examined the localization of Phosphotyrosine-p130Cas (PY-Cas) in the developing spinal cord and DRG (Fig. 2g–i). PY-Cas is present in the ventral funiculus and commissural axons in close proximity to the midline (Fig. 2g,h). Interestingly, PY-Cas is also enriched in the DREZ, DRG central projections and dorsal funiculus (Fig. 2g,i). Therefore, *Cas* mRNA, p130Cas protein, and phospho-tyrosine-p130Cas expression patterns are consistent with Cas involvement in DRG and commissural axon guidance.

**Figure 3:**
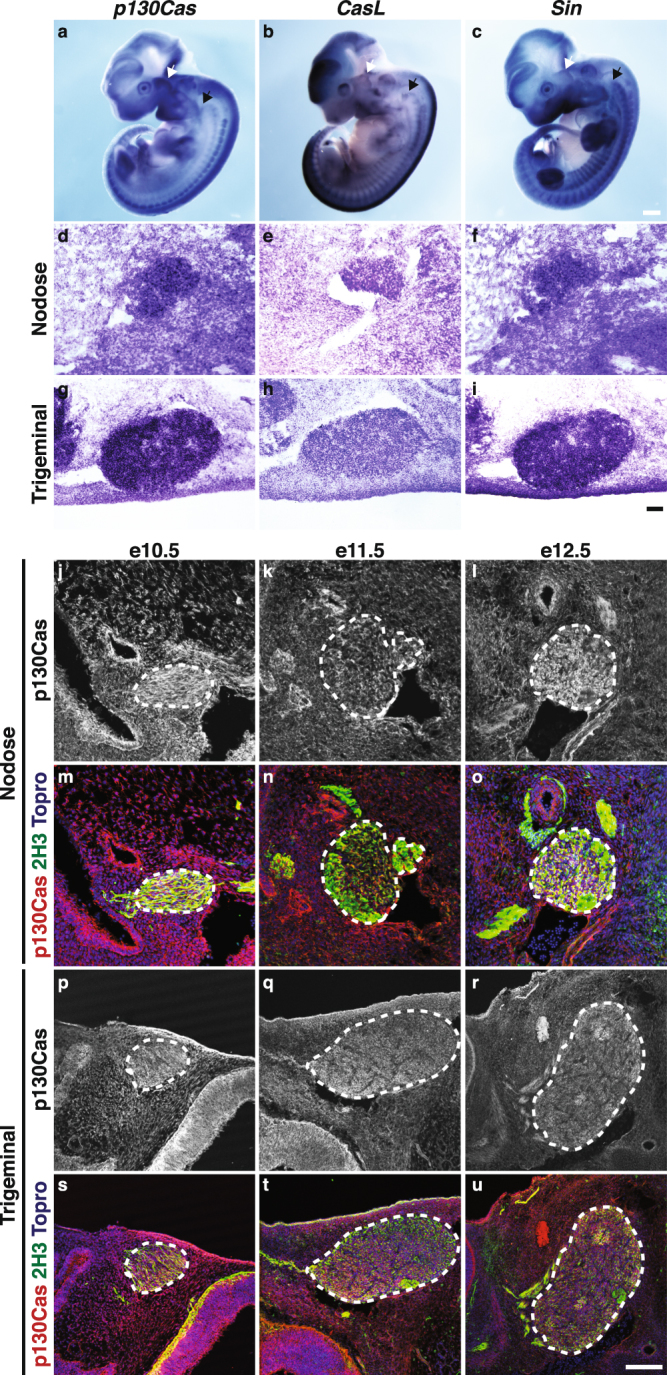
Expression of Cas mRNA and protein in trigeminal and nodose ganglia. **(a**–**c)** Whole-mount *in situ* hybridization for p*130Cas*, *CasL* and *Sin* in e11.5 embryos. White and black arrows mark the trigeminal ganglion and the nodose/petrosal complex, respectively. **(d**–**i)** *In situ* hybridization on transverse sections through the nodose (**d**–**f**) and trigeminal (**g**–**i**) of e11.5 embryos with probes against p*130Cas* (**d**,**g**), *CasL* (**e**,**h**) and *Sin* (**f**,**i**). (**j**–**u**) Expression profile of p130Cas protein (red) in transverse sections through the nodose (**j**–**o**) and trigeminal (**p**–**u**) ganglia at various developmental stages. Anti-neurofilament (2H3, green) was used to visualize axons and ToproIII (blue) was used to counterstain nuclei. Dotted lines delineate the ganglia. Scale bars: 500 μm for (**a**–**c**), 100 μm for (**d**–**f**), 200 μm for (**g**–**i**), 75 um for (**j**–**o**) and 150 um for (**p**–**u**).

**Figure 4:**
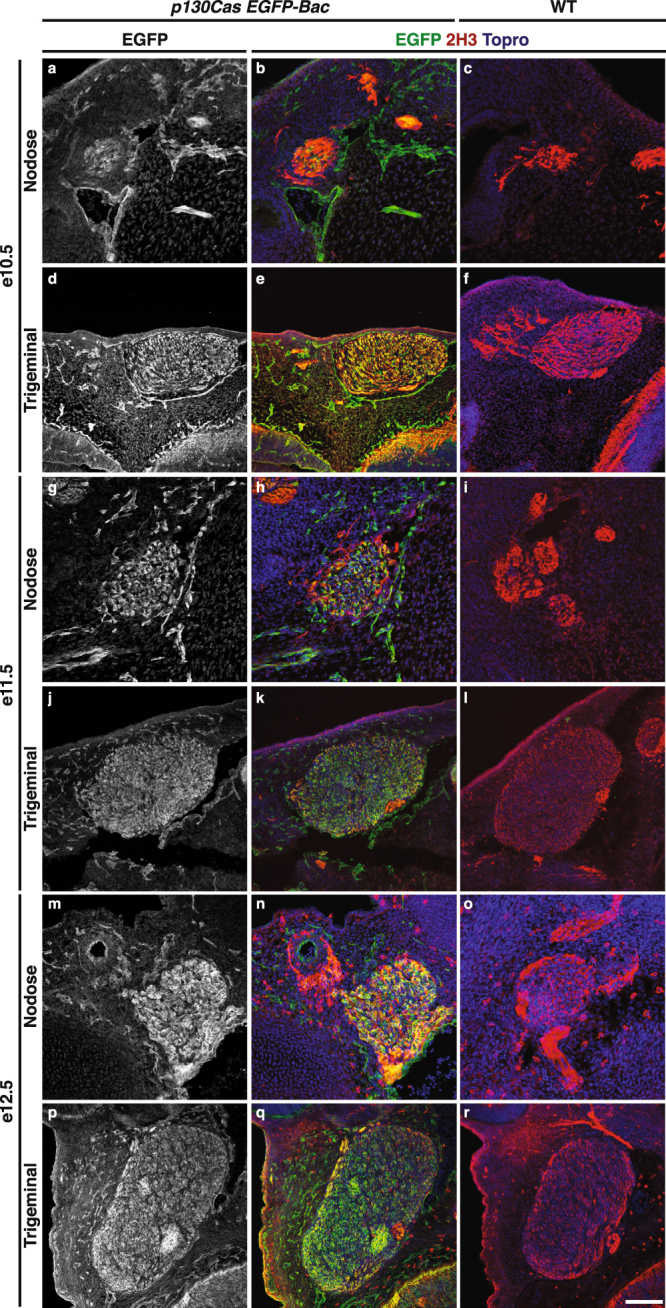
Expression analysis of *p130Cas EGFP-Bac* in cranial ganglia. (**a**–**r**) Immunofluorescence for EGFP (green) and 2H3 neurofilament (red) on transverse sections from *p130Cas EGFP-Bac* (**a**,**b**,**d**,**e**,**g**,**h**,**j**,**k**,**m**,**n**,**p**,**q**) and WT (**c**,**f**,**i**,**l**,**o**,**r**) embryos, through the nodose (**a**–**c**,**g–i**,**m**–**o**) and trigeminal (**d**–**f**,**j**–**l**,**p**–**r**) ganglia. ToproIII (blue) was used to counterstain nuclei. Note that *p130Cas*-driven EGFP expression is high throughout both ganglia. Scale bar: 75 um for (**a**–**c**,**g**–**i**) and (**m**–**o**); 150 um (**d**–**f**,**j**–**l**) and (**p**–**r**).

In addition to expression of Cas genes in the SC and DRG, whole-mount *in situ* hybridization revealed the presence of *Cas* transcripts in the trigeminal ganglion and the nodose/petrosal complex at e10.5 and e11.5 (Figs 1a–c, 3a–c). *In situ* hybridization on sections confirmed that *Cas* genes are broadly expressed in the trigeminal and nodose at e11.5 (Fig. 3d–i). Consistent with these results, p130Cas protein is found in cell bodies and projections of both ganglia from e10.5 to e12.5 (Fig. 3j–u), and overlaps with *p130Cas* mRNA expression at e11.5 (Fig. 3d,g). Expression of EGFP in *p130Cas-EGFP-Bac* animals confirmed the strong expression of *p130Cas* in trigeminal and nodose ganglia from e10.5 to e12.5 (Fig. 4a–r). No EGFP expression was detected in WT nodose or trigeminal ganglia (Fig. 4c,f,i,l,o,r).

**Figure 5:**
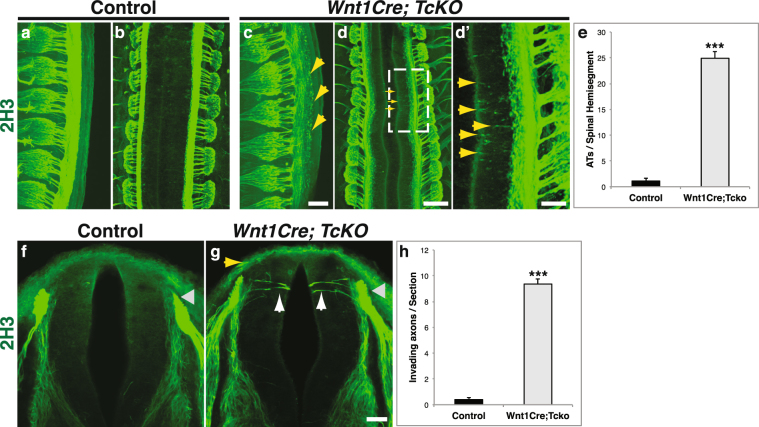
Cas adaptor proteins are required for the fasciculation of DRG central projections. **(a**–**d’)** Whole embryo immunostaining for neurofilament (2H3, green) at e12.5, from a side view (**a**,**c**) or a dorsal view (**b**,**d**,**d**’). d’ shows a higher magnification view of the dotted area in d. The centrally projecting DRG axons are severely defasciculated as they enter the spinal cord (yellow arrows). n = 6 per gentoype; presented phenotypes displayed 100% penetrance. **(e)** Quantification of free axon terminals (ATs) per spinal hemisegment, visualized from the side. Two-tailed t-student test ***p = 9.05e-24, 3–5 thoracic segments per animal, 6 animals for each genotype. **(f**–**g)** Transverse vibratome sections through e11.5 Control (**f**) and *Wnt1Cre; TcKO* (**g**) spinal cords at forelimb level stained using an antibody against neurofilament (2H3). Sensory axons invade the spinal cord gray matter prematurely in *Wnt1Cre; TcKO* animals (**g**, white arrows). Gray arrowheads: DREZ. **(h)** Quantification of number of axons invading the spinal cord per section. Two-tailed t-student test ***p = 3.82e-26. 5 sections per animal, 5 animals for each genotype. Error bars represent SEM. Scale bars: 100 μm for (**a**,**c**); 200 μm (**b**,**d**); 66.7 μm for (**d**’); and 50 μm for (**f**,**g**).

Given the expression and phosphorylation pattern of Cas adaptor proteins in the developing spinal cord and DRG, we next asked whether *Cas* genes are required for DRG and commissural axon pathfinding. Since the expression patterns of *Cas* genes during spinal cord development are highly overlapping and Cas adaptor proteins act redundantly during retina development^13^, we concurrently ablated all *Cas* genes from the dorsal spinal cord and DRG (dSC + DRG). Using *Wnt1-Cre2* mice that expresses Cre recombinase in the dorsal spinal cord and neural-crest derived structures^19^ (Supplementary Figs S4 and S5), we ablated a conditional allele of *p130Cas* in a *CasL*^−/−^*; Sin*^−/−^ double null mutant genetic background (we refer to *p130Cas*^*f/Δ*^; *CasL*^−/−^; *Sin*^−/−^ mice as triple conditional knock-outs: “TcKO”)^13^. We first confirmed the removal of functional Cas proteins by performing immunostaining for PY-Cas at e12.5: PY-Cas was almost completely absent in *Wnt1Cre; TcKO* SCs and DRGs (Supplementary Fig. S4i,j). We next examined the overall projection pattern of DRG axons in *Wnt1Cre; TcKO* and control littermates. For this and all subsequent experiments *p130Cas* ^*f/+*^; *CasL*^−/−^; *Sin*^−/−^ embryos were used as controls. In control embryos, axons from DRG sensory neurons bifurcate and project along the anterior-posterior axis of the dorsal spinal cord as a tightly fasciculated bundle, as revealed by whole-mount immunohistochemistry at e12.5 (Fig. 5a,b). This is in stark contrast to the DRG central projections of *Wnt1Cre*; *TcKO* embryos, which are highly defasciculated (Fig. 5c–d’), resulting in a highly significant increase in the number of “free” axon terminals in the dorsal SC (Fig. 5e; two-tailed t-test p = 9.06e-24). All other combinations of *Cas* family alleles display no overt phenotypes in DRG or other PNS axon tract guidance (data not shown). Interestingly, some of the defasciculated axons in *Wnt1Cre; TcKO* embryos appear to project towards the ventricular zone (Fig. 5b,d,d’). These phenotypes observed in *Wnt1Cre; TcKO* embryos are 100% penetrant (n = 6).

To further explore the role of Cas proteins during DRG axon pathfinding, we examined in more detail the innervation of the SC gray matter by sensory axons in control and *Wnt1Cre;TcKO* mutants. DRG afferent axons project to the DREZ and then stall during a “waiting period” before innervating the spinal cord. In the mouse this period extends from e11 until e13.5 for proprioceptors, or e15 for nociceptors^20,21^. In control e11.5 embryos, no sensory axons are detected medial to the DREZ and dorsal funiculus (Fig. 5f). However, there is a significant increase in the number of DRG axons that invade the gray matter of *Wnt1Cre;TcKO* embryos prematurely (Fig. 5g,h; two-tailed t-test p = 3.82e-26). This suggests that Cas adaptor proteins are required for proper fasciculation of DRG axons at the dorsal funiculus, as well as preventing these axons from entering the SC gray matter prematurely.

**Figure 6:**
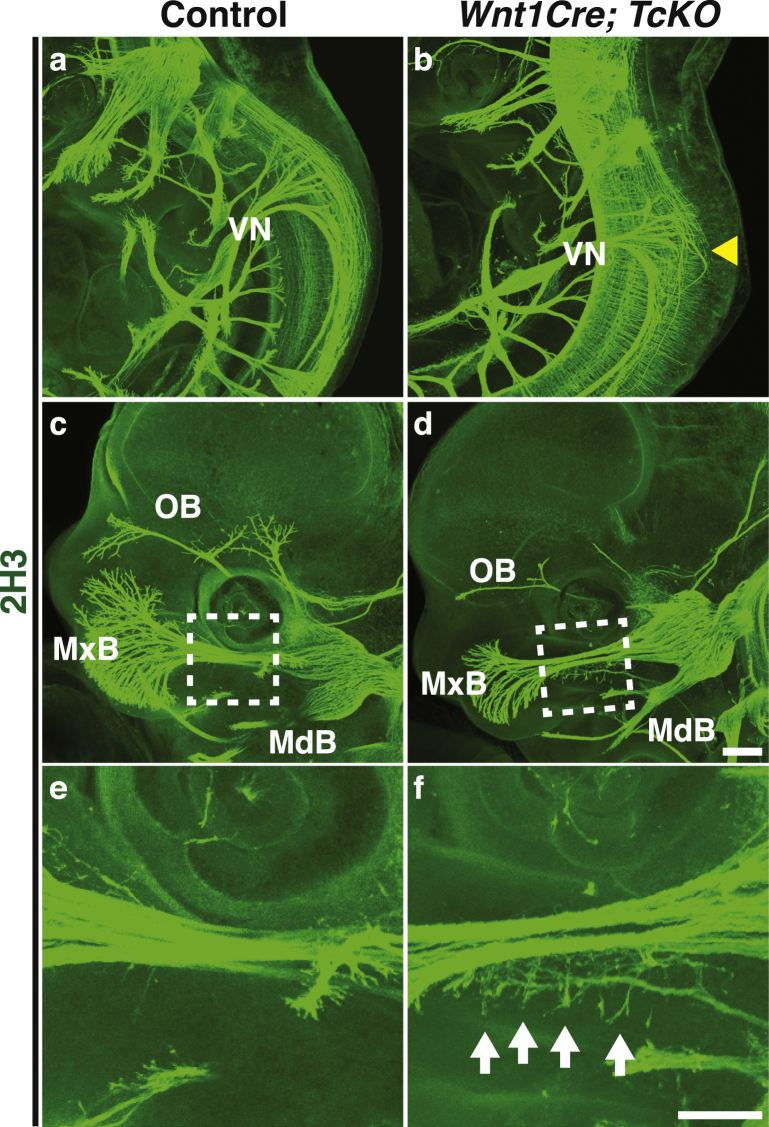
Cas adaptor proteins are essential for cranial nerve development. **(a**–**f)** Whole-mount immunostaining of Control (**a**,**c**,**e**) and *Wnt1Cre; TcKO* embryos (**b**,**d**,**f**) at e11.5 (**a**,**b**) and e12.5 (**c**–**f**). The central projections of the vagal nerve (VN) are severely defasciculated in *Wnt1Cre; TcKO* embryos (**b**, yellow arrowhead). The ophthalmic branch trigeminal nerve appears underbranched in *Wnt1Cre; TcKO* (**d**) embryos compared to controls (**c**). (**e**–**f**) Higher magnification view of white boxes in c and d reveals exuberant defasciculation of the maxillary branch of the trigeminal nerve in *Wnt1Cre; TcKO* embryos (**f**, white arrows). OB: Ophthalmic Branch; MxB: Maxillary Branch; MdB: Mandibular Branch. n = 6, 100% penetrance. Scale bars: 200 μm for (**a**–**d**); 100 μm for (**e**,**f**).

Since Cas proteins are required for DRG axon fasciculation and guidance, we hypothesized that Cas proteins may be required for pathfinding of other peripheral nerves. Could cranial nerves also require *Cas* gene function for proper fasciculation and guidance? Based on the strong expression of *Cas* genes in the nodose/petrosal complex and trigeminal ganglia (Figs 3, 4), we focused our attention on the vagal and trigeminal nerves. Normally, vagal nerve central projections join and fasciculate with descending axonal tracks coming from the midbrain (Fig. 6a)^22^. Interestingly, in *Wnt1Cre;TcKO*, the vagal afferents overshoot the descending midbrain tracks and display a defasciculated phenotype (Fig. 6b). In addition to the vagal nerve, the trigeminal nerve also shows a distinct phenotype in *Wnt1Cre;TcKO* animals (Fig. 6c–f). The maxillary branch is highly defasciculated in *Wnt1Cre;TcKO* (Fig. 6d,f) compared to control littermates (Fig. 6c,e), which is fully penetrant phenotype. Whereas the ophthalmic branch appears to be under-branched, this might be a result of a general developmental delay observed in *Wnt1Cre;TcKO* embryos by e12 (Fig. 6c–f): these embryos will die between e12.5 and e13. Overall, these data support a role for Cas adaptor proteins during peripheral nerve pathfinding.

**Figure 7:**
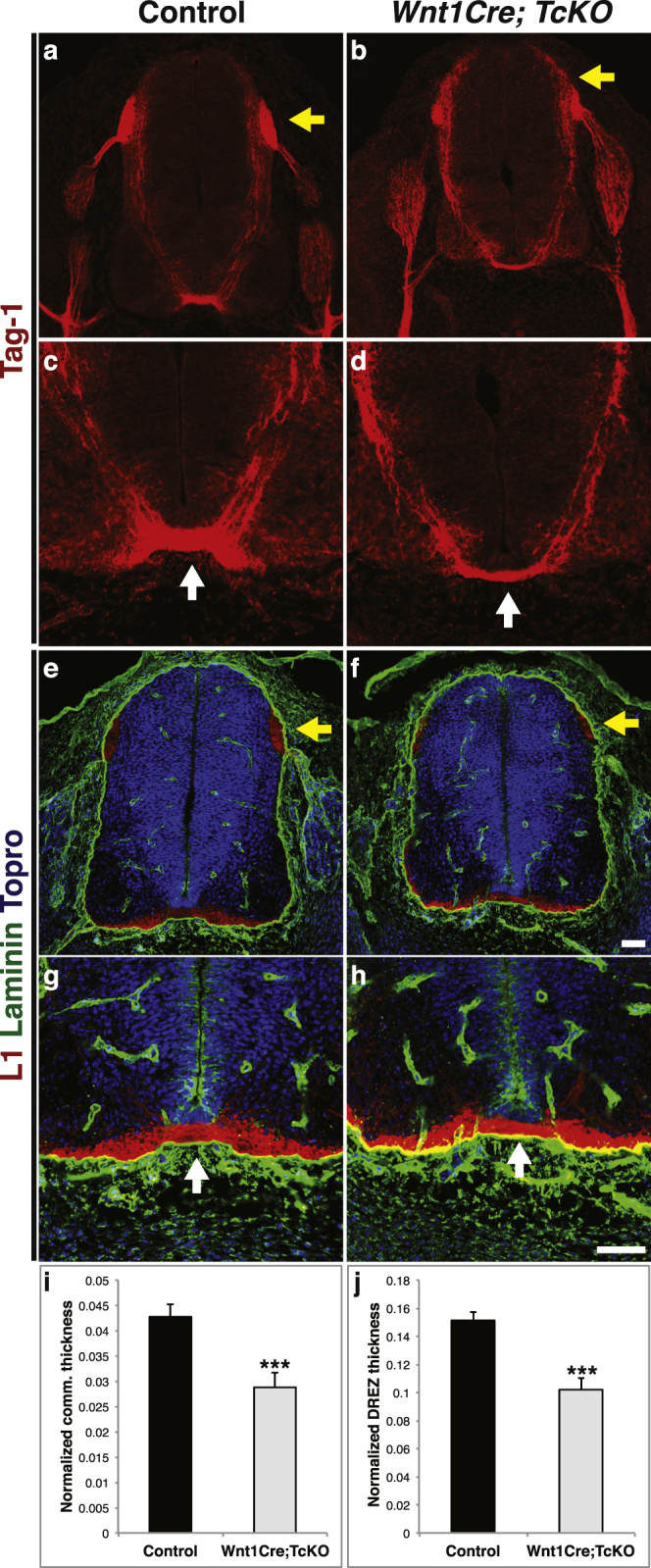
Cas mutants display a mild but significant commissural axon defect. **(a**–**h)** Transverse cryosections through control (**a**,**c**,**e**,**g**) and *Wnt1Cre;TcKO* (**b**,**d**,**f**,**h**) e11.5 spinal cords immunostained for Tag1 (**a**–**d**), L1Cam (**e**–**h**, red) and laminin (**e**–**h**, green). Sections in (**e**–**h**) were counterstained with Topro (blue). (**c**–**d**), and (**g**–**h**) are higher magnification views of (**a**–**b**), and (**e**–**f**), respectively. There is a mild reduction in the width of the ventral commissure (white arrows, **d**,**h**), and the DREZ is smaller and disorganized in *Wnt1Cre; TcKO* embryos (yellow arrows, **b**,**f**). **(i)** Quantification of the normalized commissure thickness in control and *Wnt1Cre;TcKO* embryos. Two-tailed t-student test ***p = 0.0041. 5 brachial sections per animal, 4–5 animals for each genotype. **(j)** Quantification of the normalized DREZ thickness in control and *Wnt1Cre;TcKO* embryos. Two-tailed t-student test ***p = 6.22e-5. 5 sections per animal, 5 animals for each genotype. Error bars represent SEM. Scale bars: 50 μm; Scale bar in F corresponds to (**a**,**b**,**e**,**f**); scale bar in h for (**c**,**d**,**g**,**h**).

A previous report using small interference RNA (siRNA) knock-down suggested that *p130Cas* is required for commissural axon guidance^14^. We revisited this finding by taking advantage of our complete *Cas* loss of function mouse model (Fig. 7a–j). We labeled commissural axons using the precrossing commissural axon marker Tag1 and the post-crossing marker L1^23^. A mild, yet significant reduction in the thickness of the ventral commissure was observed in *Wnt1Cre;TcKO* compared to control (Fig. 7a–i; two tailed t-test p = 0.004). This suggests that Cas genes might indeed play a conserved role in commissural axon guidance. Tag1 and L1 immunostaining also revealed a significant disorganization and reduction of the size of the DREZ in *Wnt1Cre;TcKO* animals compared to control (Fig. 7a,b,e,f,j; two tailed t-test p = 6.22e-5). These results illustrate the essential and multifaceted role of Cas proteins during both CNS and PNS circuit assembly.

Basement membrane (BM) integrity is required for proper axon guidance^22^. Thus, the abnormal fasciculation and guidance phenotypes observed at the DREZ in *Wnt1Cre;TcKO* embryos could be a secondary consequence of a disrupted basement membrane surface surrounding the spinal cord. To determine whether *Cas* genes are required for formation of the BM of the spinal cord, we analyzed its integrity in *Wnt1Cre;TcKO* animals. We visualized the BM using an antibody against laminin (Fig. 7e–h). The BM appears to be intact in *Wnt1Cre;TcKO* embryos, and is indistinguishable from control embryos (Fig. 7e–h). This suggests that *Cas* genes are dispensable for spinal cord BM formation, and that disruption of the basement membrane is unlikely to be responsible for axon pathfinding defects observed in *Wnt1Cre;TcKO* DRG central projections.

**Figure 8:**
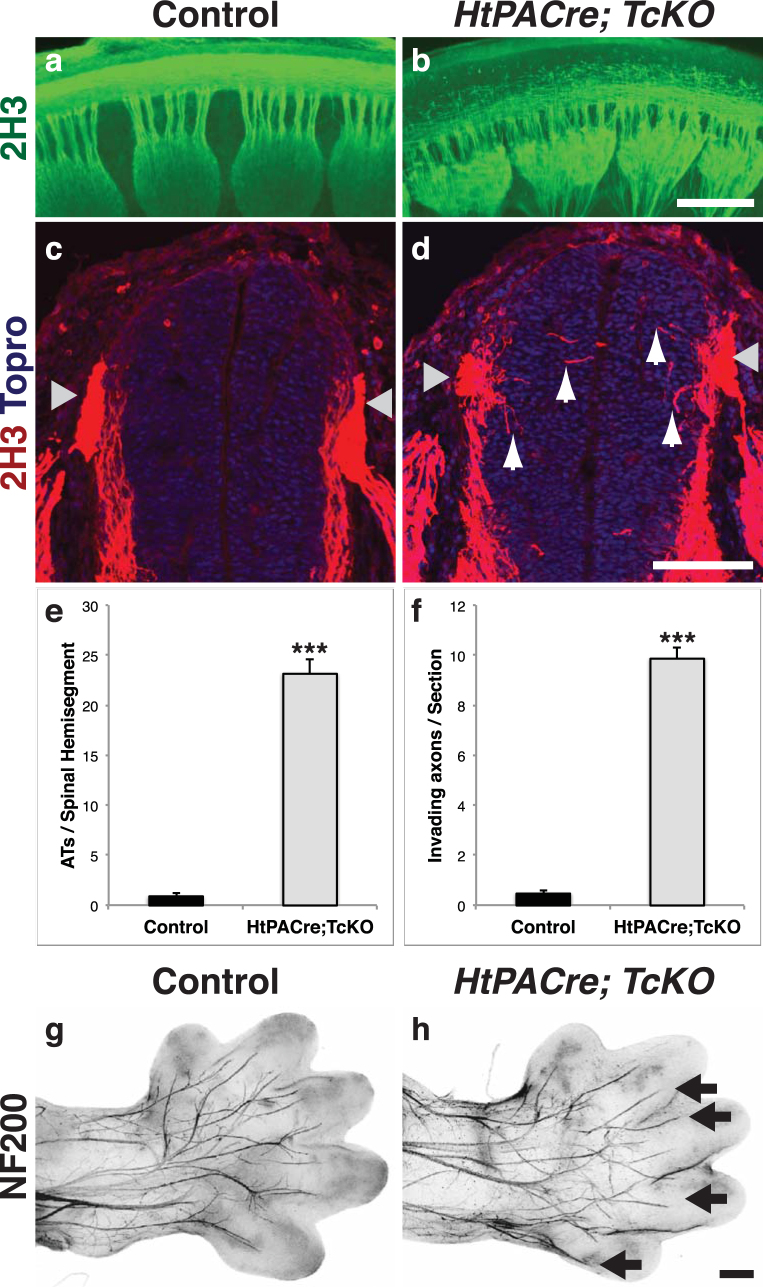
Analysis of *Cas* gene requirement in the DRG. **(a**–**b)** Wholemount neurofilament immunostaining of control (**a**) and *HtPACre; TcKO* embryos (**b**). Sideview of e12.5 spinal cords stained with 2H3. The defasciculation of DRG central projections observed in *HtPACre; TcKO* embryos (**b**) resembles that of *Wnt1Cre; TcKO* embryos (Fig. 5). (**c**–**d**) Transverse vibratome sections through e11.5 Control (**c**) and *HtPACre; TcKO* (d) spinal cords at thoracic level stained using an antibody against neurofilament (2H3, red). Nuclei were counterstained with ToproIII (blue). Sensory axons prematurely invade the spinal cord gray matter of *HtPACre; TcKO* animals (**d**, white arrows). Gray arrowheads: DREZ. **(e)** Quantification of free axon terminals (ATs) per spinal hemisegment, visualized from the side. Two-tailed t-student test ***p = 1.26e-19, 4–5 thoracic segments per animal, 5 animals for each genotype. **(f)** Quantification of number of axons invading the spinal cord per section. Two-tailed t-student test ***p = 1.76e-25. 5 thoracic sections per animal, 5 animals for each genotype. Error bars represent SEM. **(g**–**h)** Dorsal whole-mount view of e14.5 limbs stained with NF200. Select axonal branches that innervate the digits appear hyperfasciculated in *HtPACre; TcKO* (**h**) hindlimbs (black arrows). Fluorescent images were inverted to facilitate visualization. n = 6 limbs per genotype. Scale bars: 200 μm for (**a**,**b**); 100 μm for (**c**,**d**), and 250 μm for (**g**,**h**).

Selective ablation of *Cas* genes from the dorsal spinal cord and neural-crest derived PNS ganglia results in axon fasciculation and guidance defects (Figs 5 and 6); is there a DRG-autonomous requirement for *Cas* genes during axon pathfinding? To answer this question we took advantage of a transgenic line that expresses Cre recombinase under control of the human tissue plasminogen activator promoter (Ht-PA). This *HtPACre* transgene is expressed in migratory neural crest cells and their derivatives, including DRG, trigeminal and nodose/petrosal ganglia^24^, but not in the dorsal neural tube (Supplementary Figs S4 and S5). Consistent with the expression pattern of this Cre line (Supplementary Fig. S4e–h), *HtPACre*-mediated ablation of *Cas* genes in *HtPACre*; *TcKO* embryos results in a notable reduction in PY-Cas signal in the DRGs and DREZ (Supplementary Fig. S4i,k). PY-Cas can be still detected in commissural axons, and the ventral and lateral funiculus of *HtPACre*; *TcKO* embryos (Supplementary Fig. S4i,k). We examined the DRG central and peripheral projections of control and *HtPACre*; *TcKO* embryos by whole-mount and section neurofilament immunofluorescence (Fig. 8a–f). Interestingly, *HtPACre*; *TcKO* animals (Fig. 8b) display aberrant defasciculation of DRG central projections compared to control littermates (Fig. 8a,e; two-tailed t-test p = 1.26e-19). In addition, *HtPACre*; *TcKO* DRG axons invade the spinal cord gray matter prematurely (Fig. 8c,d,f; two-tailed t-test p = 1.76e-15). These abnormal defasciculation and pathfinding phenotypes look strikingly similar to those of *Wnt1Cre;TcKO* embryos (Fig. 5). However, *HtPACre*; *TcKO* vagal and trigeminal nerve projections look indistinguishable from controls (data not shown). This could be partially explained by the low level of recombination driven by the *HtPACre* transgene in these ganglia (Supplementary Fig. 5).

The stereotyped innervation pattern of the limb by sensory axons provides an excellent model to analyze DRG peripheral projection branching and fasciculation^25,26^. The early lethality of *Wnt1Cre;TcKO* animals (between e12 and e13) precluded us from performing analysis of limb innervation in those animals. Because *HtPACre*; *TcKO* animals survive at least until early adulthood, we explored hind-limb innervation in *HtPACre*; *TcKO* e14.5 embryos and control littermates using neurofilament 200 (NF200), a marker for mechanosensory aβ fibers (Fig. 8g,h). The innervation pattern in *HtPACre*; *TcKO* hind-limbs is abnormal compared to control limbs (Fig. 8g,h). Mechanosensory fibers stall prematurely and hyper-fasciculate in *HtPACre*; *TcKO* animals. The DRG-specific deletion of *p130Cas* in a *CasL*^−/−^*; Sin*^−/−^ background is suggestive of a DRG-autonomous role for *Cas* genes during the guidance and fasciculation of somatosensory peripheral and central projections.

**Figure 9:**
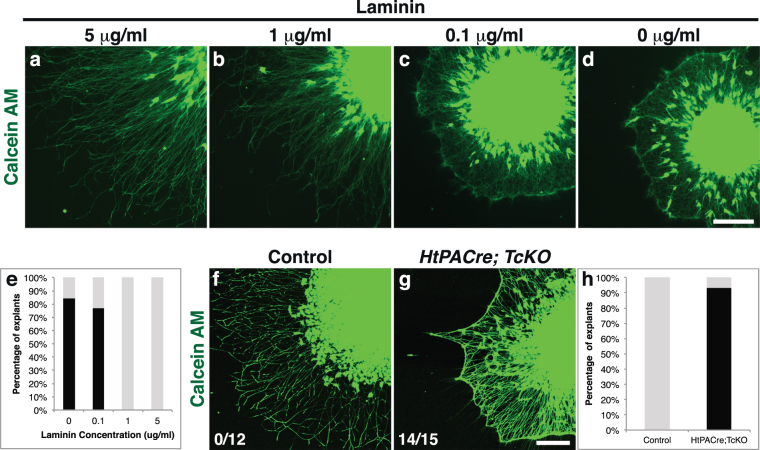
*Cas* genes are required for normal fasciculation *in vitro*. (**a**–**d)** e13.5 DRG explants from WT animals cultured on 100 μg/ml poly-D-lysine plus different concentrations of laminin. Cells were visualized using Calcein-AM. **(e)** Quantification of *in vitro* DRG phenotypes presented as percentage of explants that display webbing (black bars) vs. percentage of explants with normal morphology (gray bars). There is a significant difference in the webbing percentages under different culture conditions (Freeman-Halton extension of the Fisher exact probability test, p = 4.94e-8; 9–16 explants for each condition). **(f**–**g)** DRG explants from control (**f**) and *HtPACre; TcKO* (**g**) embryos cultured on 100 μg/ml poly-D-lysine and 5 μg/ml laminin. Cells were visualized using Calcein-AM. The proportion of explants from each genotype displaying webbing is shown at the lower left corner of each panel. *HtPACre; TcKO* explants display an abnormal “cobweb” morphology (**g**), similar to WT explants cultured on poly-D-lysine alone (**d**). **(h)** Percentage of explants that display webbing (black bars) vs. percentage of explants with normal morphology (gray bars). The difference in the proportion of WT and *HtPACre; TcKO* explants that display the cob-web phenotype is highly significant (two-tailed Fisher exact test, p = 7.47e-7; 12–15 explants for each genotype). Scale bars 200 μm.

Based on our results, we hypothesized that Cas adaptor proteins might regulate fasciculation at the DREZ by allowing axons to sense and adhere to the extracellular substrate. This ability to sense the environment will be critical at choice-points like the DREZ, where axons must distinguish between adhesion to the extracellular matrix (ECM) and to other axons to decide whether to join an axonal tract or not^27^. We first set out to ask whether altering the ECM environment results in changes to the fasciculation properties of DRG axons. We established a simple model to answer this question: we cultured e13.5 DRG explants on a set concentration of Poly-D-lysine (0.1 mg/ml) and a variable concentration of the ECM protein laminin (from 0 μg/ml to 5 μg/ml) (Fig. 9a–d). DRG axons plated on 5 μg/ml and 1 μg/ml laminin grew radially and display a characteristic sun-like morphology. Interestingly, when cultured on low (0.1 μg/ml) or no laminin, e13.5 DRG-explant axons fasciculate together forming a rim of axon bundles at a distance from the explant (Fig. 9c,d). This “cob-web” phenotype is observed in the great majority of explants cultured with low or no laminin (76.9% and 84.2%, respectively), but is never observed in explants cultured on 1 μg/ml or 5 μg/ml (Fig. 9a–e; Freeman-Halton extension of Fisher exact probability test, p = 4.94e-8). The overall growth of DRG axons is also affected for explants grown on low- or no-laminin, as compared to axons grown on 5 μg/ml laminin (Supplementary Fig. S6a; One-Way Anova, p = 1.11e-16; Tukey HSD post-hoc test p < 0.00001 for both pairwise comparisons). These results suggest that a change to the ECM composition can dramatically affect the fasciculation preferences and growth rate of DRG axons.

To investigate how Cas adaptor proteins participate in the fasciculation of sensory axons, we cultured DRG explants from control and *HtPACre*; *TcKO* on 5 μg/ml laminin. Control DRG explants axons grew radially in a sun-like pattern, as described above (Fig. 9f). Interestingly, *HtPACre*; *TcKO* axons fasciculate together displaying the cob-web morphology (Fig. 9g,h). *HtPACre*; *TcKO* axons also display a reduced level of growth as compared to control (Supplementary Fig. S6b; two tailed t-test, p = 1.49e-7). The webbing phenotype was not observed in control DRG explants under the same conditions (Fig. 9f,h; Fisher Exact Probability test two-tailed p = 7.47e-7), but is indistinguishable from the phenotype observed in WT explants cultured on no laminin (Fig. 9d,e,g,h; Fisher Exact Probability test one-tailed p = 0.397; two-tailed p = 0.613). This suggests that *HtPACre*; *TcKO* axons cultured on 5 μg/ml laminin behave as if there was no laminin in the environment. Importantly, the fact that *HtPACre; TcKO* DRG axons display a fasciculation phenotype in an isolated *in vitro* setting reinforces the idea of a DRG-autonomous function of *Cas* genes during DRG fasciculation. Overall, our data support a model whereby Cas proteins regulate DRG axon fasciculation *in vitro* and *in vivo* by allowing axons to sense the ECM.

## Discussion

Here, we demonstrate an evolutionarily conserved requirement for Cas adaptor proteins during guidance and fasciculation of PNS axons. Our results in the mouse are consistent with the previously described role of *dCas* in Drosophila PNS development^15^. In Drosophila, Cas phosphorylation and function during PNS axon fasciculation and guidance is mainly regulated by integrins. Similarly, during mammalian retina migration, integrin-β1 appears to be the primary regulator of Cas function, as shown by the identical phenotype in their respective null mutants, and molecular epistasis results^13^. Whereas the peripheral innervation phenotypes in the developing limb are similar in *HtPACre; Itgb1*^*f/f*^ and *HtPACre; TcKO* mutants, the severe central projection phenotype observed in *Cas TcKO* mice was not observed in their *integrin-β1* counterparts^25^. This suggests that integrin-β1 is not the sole upstream regulator of Cas adaptor function during DRG central projection pathfinding. Whether integrins act redundantly to regulate Cas function or a different guidance cue- or adhesion-receptor is involved in this process remains to be investigated.

In addition to DRG pathfinding defects, *Wnt1Cre;TcKO* animals display aberrant trigeminal and vagal nerve fasciculation phenotypes. While the vagal nerve phenotype is very specific and fully penetrant, the under-branching trigeminal phenotypes could be likely attributed to a pleiotropic delay in embryonic development. Interestingly, *HtPACre*; *TcKO* vagal nerve central projections look indistinguishable from controls. The most plausible explanation for the lack of an abnormal phenotype in these mutants is that the *HtPACre* transgene is a poor driver of recombination in the nodose/petrosal complex (Supplementary Fig. S5). Whereas *Wnt1Cre* expression results in strong recombination in both nodose and trigeminal, *HtPACre* drives recombination in a very low number of nodosal cells (Supplementary Fig. S5). Given this caveat, we cannot confirm or exclude the possibility that Cas genes might act in a cell-autonomous manner during the fasciculation of vagal projections.

In regards to Cas function during commissural axon guidance, it was reported that *p130Cas* mediates Netrin signaling during this pathfinding event^14^. The single *Wnt1Cre*; *p130Cas*^*f/f*^ mutants displayed no overt axon guidance phenotypes (data not shown). The complete *Cas* loss-of-function mouse model (*Wnt1Cre;TcKO*) did show a significant thinning of the commissure by e11.5, although the phenotypes observed in the chicken knock-down experiments were much more striking^14^. Furthermore, the observed phenotype was notably milder than that of the *Netrin*^−/−^ mice, which have almost no detectable ventral commissures^28^. This result suggests that if *Cas* genes indeed act downstream of Netrin during mouse commissural axon guidance, they would more likely serve as modulators than obligate-downstream effectors. Another possibility is that *Cas* genes might play a more general role during commissural axon fasciculation.

An unexpected discovery was the fact that *Cas-null* DRG axons displayed a different growth pattern on laminin than control explants, re-fasciculating with each other at a distance from the somas (Fig. 9). *Cas-null* DRG axons behave as if there was no laminin in the extracellular environment, even when cultured on laminin. This result raises the intriguing possibility that *Cas* genes are required for neurons to distinguish between secreted adhesion molecules in the ECM (e.g. laminin) and neural adhesion molecules present in axons themselves. This environmental assessment will be particularly important at choice-points like the DREZ, and could offer a potential mechanism underlying the DRG central projection defasciculation phenotypes observed in *HtPACre*; *TcKO* and *Wnt1Cre;TcKO* embryos. Alternatively, Cas might be important for DRG axons to pause at the DREZ to sense repulsive and attractive cues on their way to finding their targets. In this regard it is interesting to note that some of the sensory phenotypes observed in *HtPACre*; *TcKO* and *Wnt1Cre;TcKO* embryos resemble aspects of *Robo/Slit*^29,30^, *dystroglycan*^22^, *netrin*^31^, and *Neuropilin-1*^20^ mutants. Future studies will investigate the role of Cas adaptor proteins during the interplay between adhesion to the substrate and guidance cue signaling.

## Materials and Methods

### Animals

The day of vaginal plug observation was designated as embryonic day 0.5 (e0.5) and the day of birth postnatal day 0 (P0). Control animals for all experiments were *p130Cas* ^*f/*+^; *CasL*^−/−^; *Sin*^−/−^. Generation of the *HtPACre*, *Wnt1Cre*, *p130Cas*^*f/f*^, *CasL*^−/−^ and *Sin*^−/−^ transgenic mouse lines has been described previously^13,19,24,32,33^. All animal procedures presented here were performed according to the University of California, Riverside’s Institutional Animal Care and Use Committee (IACUC) guidelines. All procedures were approved by UC Riverside IACUC.

### *In situ* Hybridization

*In situ* hybridization was performed on spinal cord frozen sections (20 μm thickness) using digoxigenin-labeled cRNA probes, as previously described^34^. Whole-mount RNA *in situ* hybridization was performed as described^35^. Generation of the *p130Ca*s, *CasL* and *Sin* cRNA probes has been described in^13^.

### Immunofluorescence

Mice were perfused and fixed with 4% paraformaldehyde for 1 hour to O/N at 4 °C, rinsed, and processed for whole-embryo staining or sectioned on a vibratome (75 µm). Whole-mount immunofluorescence was performed as described in^36^. Immunohistochemistry of floating sections was carried out essentially as described^37^. For cryostat sections, following fixation, embryos were equilibrated in 30% sucrose/PBS and embedded in OCT embedding media (Tissue-Tek). Transverse spinal cord sections (20–40 µm) were obtained on a Leica CM3050 cryostat and blocked in 10% goat serum in 1 × PBS and 0.1% Triton-X100 for 1 hr at room temperature. Sections were then incubated O/N at 4 °C with primary antibodies.

Sections were then washed in 1 × PBS and incubated with secondary antibodies and TOPRO-3 (Molecular Probe at 1:500 and 1:2000, respectively). Sections were washed in PBS and mounted using vectorshield hard-set fluorescence mounting medium (Vector laboratories). Confocal fluorescence images were taken using a Leica SPE II microscope. Primary antibodies used in this study include: rabbit anti-p130Cas C terminal (Santa Cruz, 1:200), rabbit anti-p130Cas PY165 (Cell Signaling Technology, 1:100), rabbit anti-laminin (Sigma, 1:1000), rabbit anti-GFP (Lifescience Technologies, 1:500), chicken anti-GFP (AVES, 1:1000), mouse anti-Neurofilament (2h3, Developmental Studies Hybridoma Bank, 1:500), mouse anti-Tag1 (4D7, Developmental Studies Hybridoma Bank, 1:50), Rat anti-L1 (MAB5272, Millipore, 1:500) and rabbit anti-NF-200 (Millipore, 1:500).

### Quantification of spinal cord ventral commissure and DREZ thickness

Thickness of the DREZ and ventral commissure were measured on L1-immunostained cryosections at e11.5 (20-μm sections). The thickness values for the ventral commissure were normalized to the distance between roof plate and floor plate for each section, as described previously^38,39^. The maximal thickness of the DREZ for each hemi-spinal cord was recorded and normalized to the distance between the BM and the ventricular zone at the same dorso-ventral level. Thickness was measured at brachial levels. Five sections per embryo, from 3–5 embryos were analyzed. Statistical differences for mean values between two samples were determined by two-tail Student’s t-test for independent samples.

### Quantification of axons invading the spinal cord

50-μm vibratome sections were stained with neurofilament (2H3) and the number of axons entering the spinal cord were quantified. “Free” axon terminals per segment were quantified using high magnification images of cleared whole-mount embryos immunostained for 2H3. Briefly, cleared embryos were placed on their side in a transparent glass well filled with 2 parts Benzyl Benzoate: 1 part Benzyl Alcohol (BABB). The side of the embryo facing upwards was imaged under a 20× objective by collecting confocal optical sections until the spinal cord midline was reached. Z-stack images, consisting of 15 to 25 images each, were then flattened by using the Max Intensity Projection feature of Image-J. The number of free axon terminals was determined by counting free terminals from the anterior end of a DRG to the anterior edge of the following DRG. Statistical differences for mean values between two samples were determined by two-tail Student’s t-test for independent samples.

### Tissue Culture

DRGs from e13.5 embryos were dissected in ice-cold L15 (Invitrogen). DRG explants were then plated on acid-washed glass coverslips previously coated with 0 to 5 μg/ml laminin and 100 μg/ml polyD-lysine. DRGs were then cultured for 18 hours in enriched Opti-MEM/F12 media containing 15 ng/ml NGF, as previously described^40^. Live explants were stained with Calcein-AM (Invitrogen) and then imaged. For the Control vs. *HtPACre; TcKO* explant experiment a total of 12–15 explants from 3 independent experiments were analyzed.

### Availability of data and materials

All data analyzed during this study are included in this article.

### Ethics approval

All animal procedures presented here were performed according to the University of California, Riverside’s Institutional Animal Care and Use Committee (IACUC)-approved guidelines.

## Electronic supplementary material

Supplementary Information
