# Supplementary material for: Cas Adaptor Proteins Coordinate Sensory Axon Fasciculation
Source: Sci Rep. 2018 Apr 16;8:5996. doi: 10.1038/s41598-018-24261-x (PMC5902548; doi:10.1038/s41598-018-24261-x)
Supplement: Supplementary file 1 — Supplementary Information [file 41598_2018_24261_MOESM1_ESM.pdf]

Supplementary Information

Cas Adaptor Proteins Coordinate Sensory Axon Fasciculation

Tyler A. Vahedi-Hunter [a], Jason A. Estep [b], Kylee A. Rosette [c], Michael L. Rutlin [d], Kevin M. Wright [c], and Martin M. Riccomagno [a, b, *]

a. Neuroscience Program, Department of Molecular, Cell and Systems Biology, University of California, Riverside, CA 92521, USA.

b. Cell, Molecular and Developmental Biology Program, Department of Molecular, Cell and Systems Biology, University of California, Riverside, CA 92521, USA.

c. Vollum Institute, Oregon Health & Science University, Portland, OR 97239, USA.

d. Department of Biochemistry and Molecular Biophysics, Columbia College of Physicians and Surgeons, Columbia University, New York, New York 10032, USA.

Supplementary Figure Legends

Supplementary Figure S1. Expression of *Cas* mRNA in DRGs. (a-c) Transverse sections through embryonic SC and DRG stained by in situ hybridization with probes against p*130Cas* (a), *CasL* (b) and *Sin* (c) at e11.5. Dotted lines delineate the DRG. **(d-f)** Whole-mount *in situ* hybridization with sense control probes for p*130Cas*, *CasL* and *Sin* in e10.5 embryos. Scale bars: 50 µm for a-c; 500 µm for d-f.

Supplementary Figure S2. p130Cas expression in embryonic SC and DRG. (**a-d**) Expression profile of p130Cas protein (red) in transverse sections through the mouse spinal cord at e10.5 (a, b) and e11.5 (c, d). Anti-Neurofilament (2H3, green) was used to reveal axons. TοproIII (blue) was used to counterstain nuclei. White arrowheads: DREZ; Yellow arrowheads: DRG. Scale bar: 150 µm.

Supplementary Figure S3. Expression analysis of *p130Cas EGFP-Bac* in developing SC. (**a-i**) Immunofluorescence for EGFP (green) and 2H3 neurofilament (red) on transverse sections from *p130Cas EGFP-Bac* (a, b, d, e, g, h) and WT (c, f, i) spinal cords at e10.5 (a-c), e11.5 (d-f) and e12.5 (g-i). ToproIII (blue) was used to counterstain nuclei. Note that *p130Cas*-driven EGFP expression is high in DRG, dorsal SC, DREZ and ventral roots. Panels g and h are also presented in Figure 2. Scale bar: 100 µm for a-c; 200 µm for d-i.

Supplementary Figure S4. Recombination pattern of Cre lines in the spinal cord and DRG. (a-h) Cre activity as reported by tdTomato expression (red) in e12 control reporter animals (*Ai9*, a, c, e, g), *Wnt1Cre; Ai9* (b, d), and *HtPACre; Ai9* (f, h). Anti-2H3 (green) was used to visualize axons and ToproIII (blue) to label nuclei. *HtPACre; Ai9* displays some sparse labeling in the SC, which appears to be stochastic. **(i-k)** Immunostaining for PY-Cas in Control (i), *Wnt1Cre;TcKO* (j) and *HtPACre*; *TcKO* (k) e12.5 spinal cords. Note the efficient ablation of phosphorylated-Cas from DRG and DREZ in *Wnt1Cre;TcKO* and *HtPACre*; *TcKO* embryos. As expected, PY is still detected in the ventral funiculus and commissural axons of *HtPACre*; *TcKO* animals (k, white

arrowheads). Yellow arrowheads: DRG; Red arrowheads: DREZ. Scale bars: 100 μm for a-h; 200 μm for i-k.

Supplementary Figure S5. Recombination pattern of Cre lines in the nodose and trigeminal ganglion. (a-l) tdTomato expression (red) in *Wnt1Cre; Ai9* (a, b, d, e), *HtPACre; Ai9* (g, h, j, k), and *Ai9* control littermates (c, f , i ,l) at e11.5. Transverse sections through the trigeminal (a-c, g-i) and nodose (d-f, j-l) ganglia. Anti-2H3 (green) was used to label axons and ToproIII (blue) to label nuclei. *Wnt1Cre* (a, b, d, e) strongly drives recombination in the nodose and trigeminal ganglia. *HtPACre; Ai9* shows lower number of neurons expressing tdTomato in both ganglia, and appears to be a very inefficient driver of recombination in the nodose. Scale bar: 150 μm for a-c and g-i; 75 μm d-f, j-l.

Supplementary Figure S6. Axon growth of DRG explants. (a-b) Mean length of the longest neurite per explant as a measure of axon growth. (a) WT DRG axons grown on different concentrations of laminin. Axons grown on low-(0.1 μg/ml) or no-laminin grow significantly less than axons grown on 1 μg/ml or 5 μg/ml laminin (One-Way Anova p=1.1102e-16; n=15-16 explants per condition; *** Tukey HSD post-hoc test p<0.00001 vs. 0 and 0.1 μg/ml). (b) Quantification of Control and *HtPACre; TcKO* axon growth on 5 μg/ml laminin + 100 μg/ml poly-D-lysine. *HtPACre; TcKO* axons grow significantly less than control axons (*** two-tailed t-test p=1.489e-7, n=15 explants per genotype).

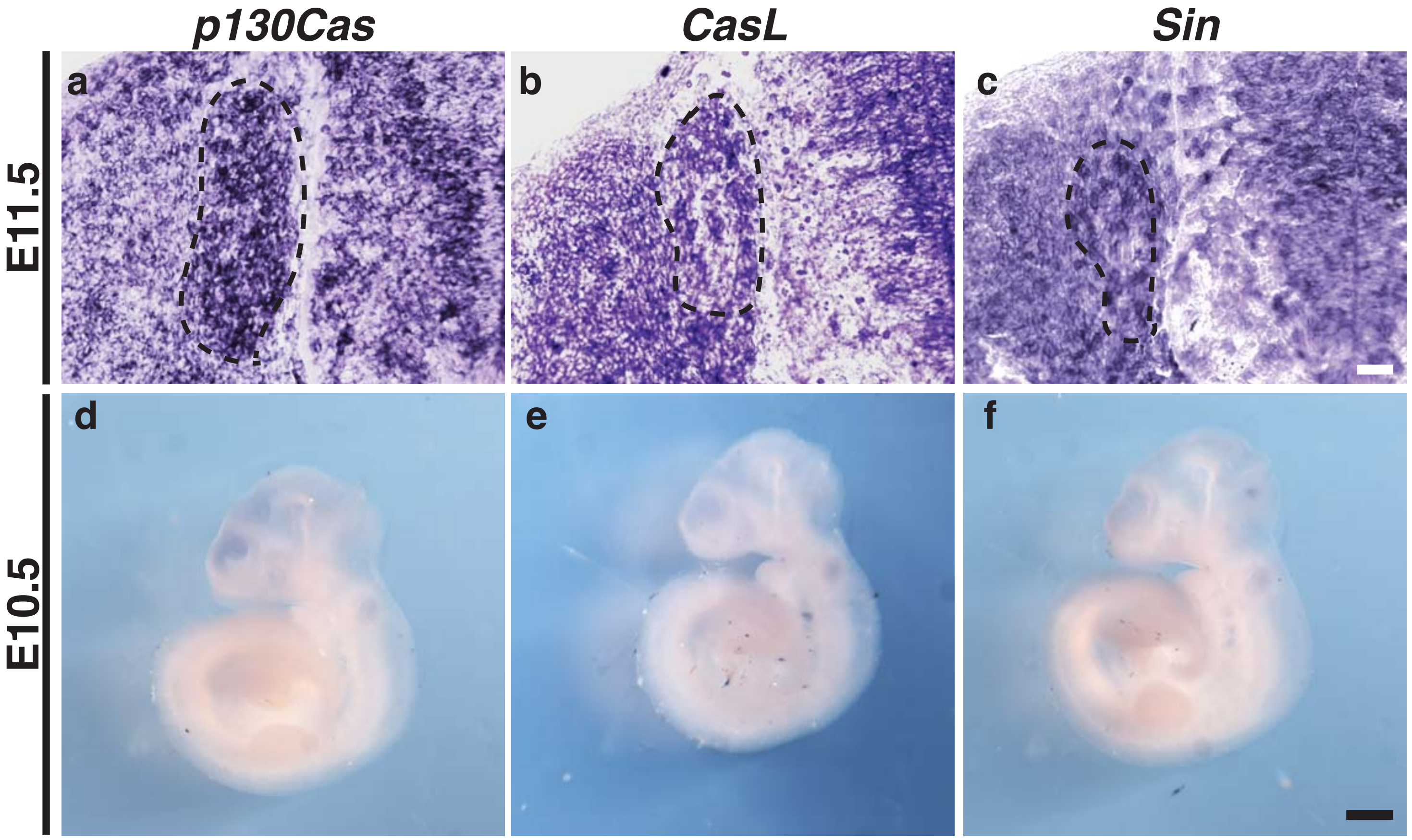

Supplementary Figure S1

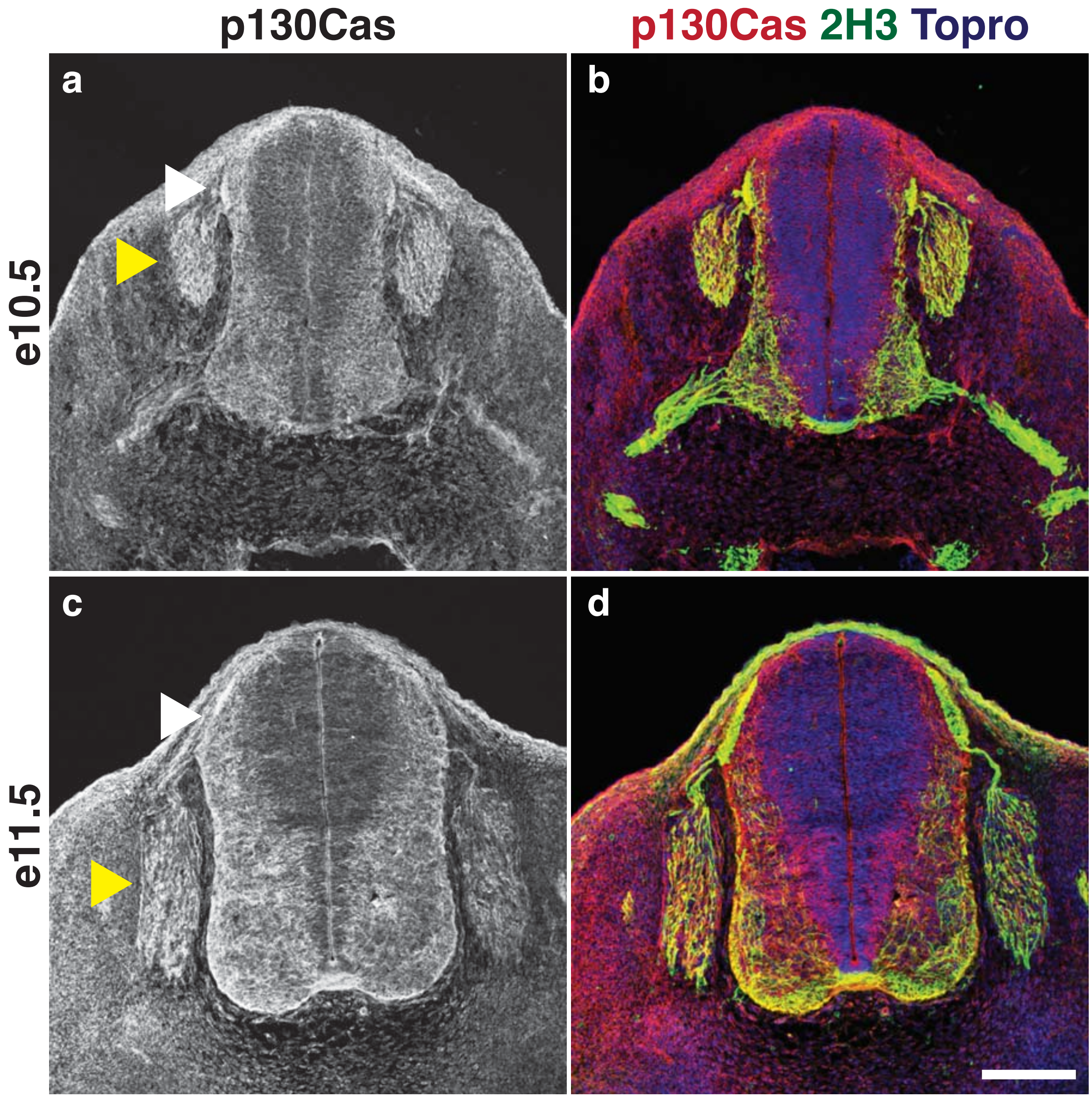

Supplementary Figure S2

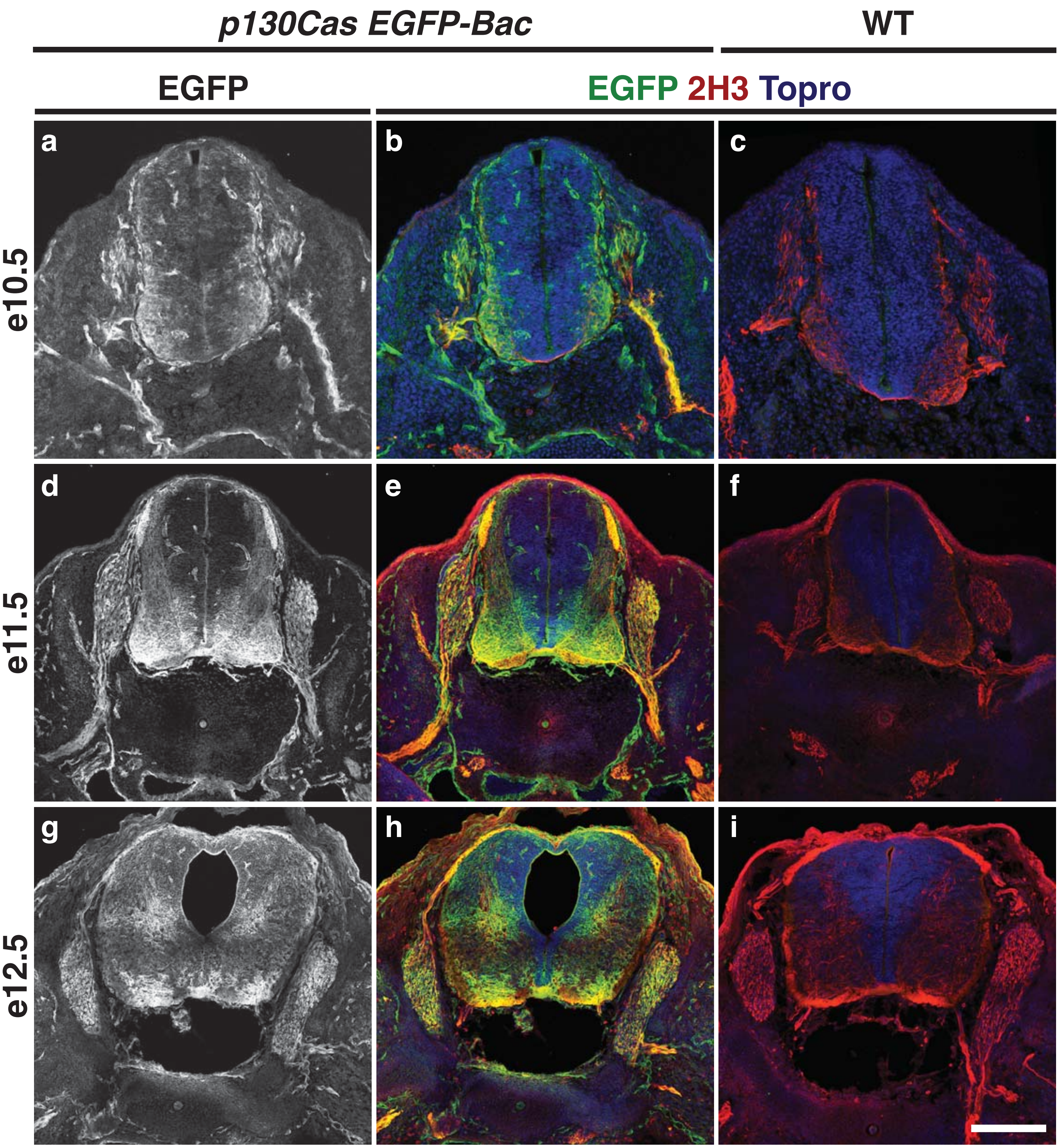

Supplementary Figure S3

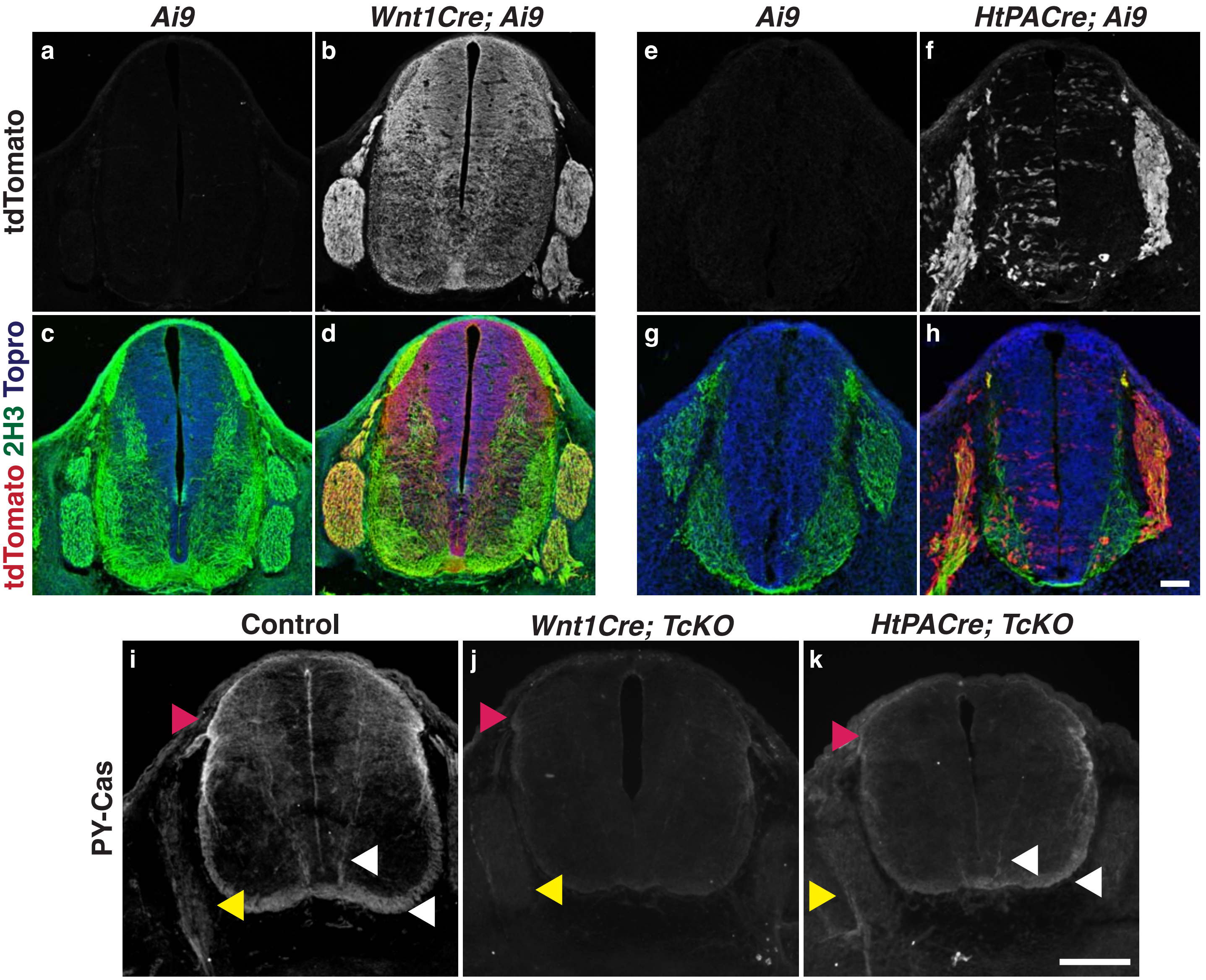

Supplementary Figure S4

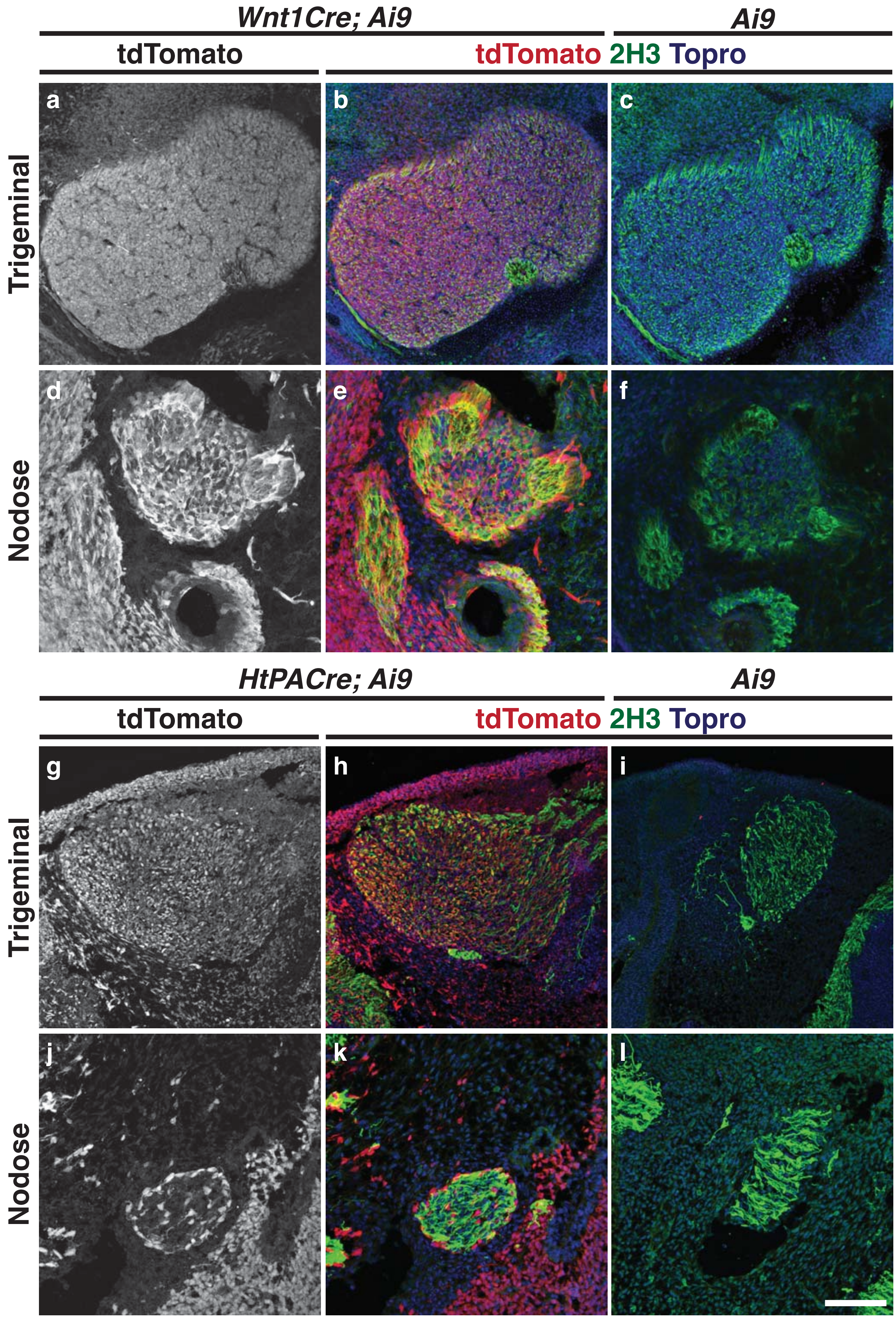

Supplementary Figure S5

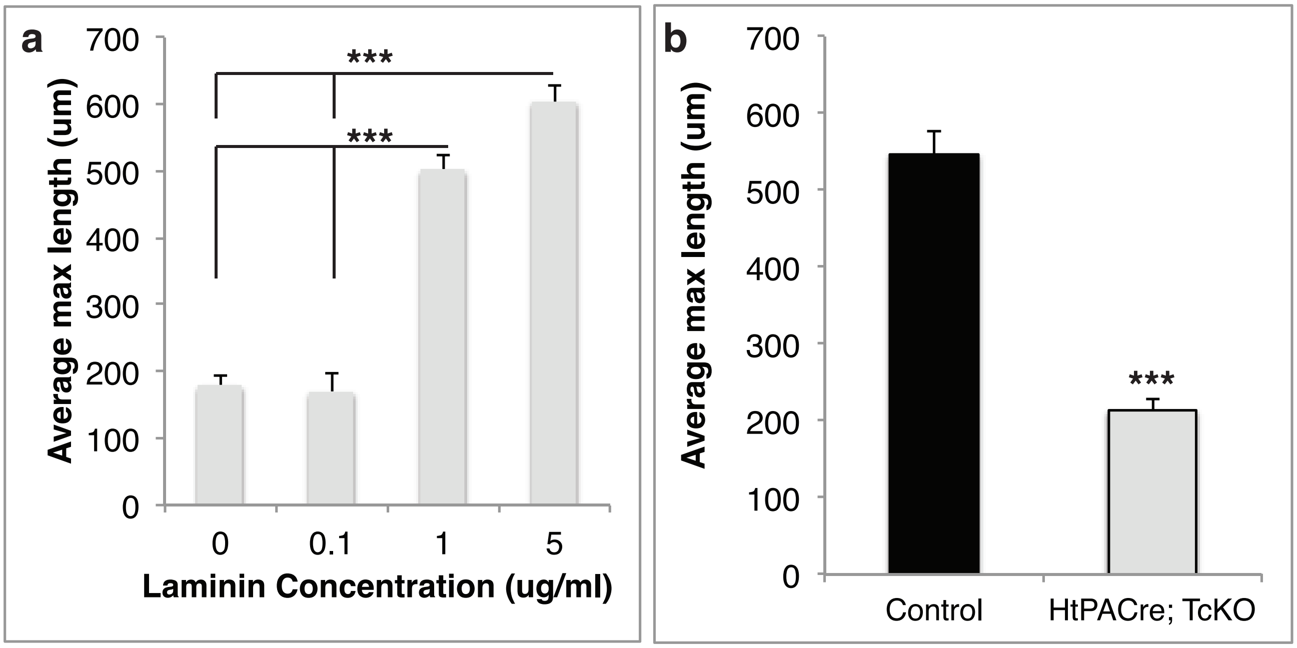

Supplementary Figure S6
